# A prophylactic TXA administration effectively reduces the risk of intraoperative bleeding during open management of pelvic and acetabular fractures

**DOI:** 10.1038/s41598-023-39873-1

**Published:** 2023-08-02

**Authors:** Guy Romeo Kenmegne, Chang Zou, Yixiang Lin, Yijie Yin, Shenbo Huang, Erandathie Lasanda Banneyake, Imani Savishka Gunasekera, Yue Fang

**Affiliations:** 1 Department of Orthopedics, West China Hospital of Sichuan University, Chengdu, 610041 China; 2 Trauma center, West China Hospital of Sichuan University, Chengdu, 610041 China

**Keywords:** Health care, Medical research, Rheumatology

## Abstract

This study aimed to evaluate the efficacy of perioperative intravenous TXA in reducing blood loss in pelvic and acetabular fracture patients managed surgically. The study included 306 consecutive patients, divided as: group I, 157 patients who did not receive perioperative infusion of TXA and group II, 149 patients who received perioperative TXA. The perioperative blood test results and complication rates were compared between the two groups. The average perioperative hematocrit was higher during the preoperative period than during the first, second and third postoperative day in both groups. In the estimated blood loss between the two groups, there was a significant difference of 1391 (± 167.49) ml in group I and 725 (± 403.31) ml in group II respectively (p = 0.02). No significant difference was seen in the total of intraoperative transfusion units as well as in the total units of blood transfused. There was a reduced level of postoperative hemoglobin (9.28 ± 17.88 g/dl in group I and 10.06 ± 27.57 g/dl in group II compared to the values obtained in preoperative investigations (10.4 ± 2.37 g/dl in group I and 11.4 ± 2.08 g/dl in group II); with a significant difference in postoperative transfusion rates (p = 0.03). Therefore, the use of TXA effectively reduces the risk of intraoperative bleeding during open management of pelvic and acetabular fractures.

## Introduction

Orthopedic surgery entails the possibility of substantial blood loss. Historically, allogeneic blood transfusions were used quite frequently to treat this blood loss. Reducing the usage of allogeneic blood has been a major effort in recent years^1,2^. Infrequent and complicated injuries like pelvic and acetabular fractures necessitate extensive orthopedic procedures. Both the injury itself and the necessary surgical operation can cause significant blood loss, with prior studies reporting total blood loss ranging from 1232 to 2818 mL^3^.

To prevent anemia and hypovolemic shock, blood transfusion is often required. However, massive transfusions can cause serious complications such as life-threatening immunologic responses, cardiovascular damage, acute lung injury, HIV and Hepatitis C transmission^4,5^. Certain previous studies on orthopedic patients revealed a significantly increased risk of postoperative infection^6–8^.

In clinical practice, several antifibrinolytic agents including tranexamic acid (TXA), aprotinin and epsilon-aminocaporic acid have been introduced with the purpose of reducing the perioperative blood loss and blood transfusion. Out of these three, TXA has been the most widely used in cardiac surgeries^9^. In recent years, TXA (Cyklokapron, Pfizer, New York, New York) an increasingly popularized synthetic derivative of lysine which competitively inhibits the conversion of plasminogen to plasmin has been widely used in trauma and arthroplasty^10–12^.

Analysis of previously published data from 26 randomized controlled trials have demonstrated that TXA was able to reduce perioperative blood loss (at least 300 mL) and reduce the transfusion rate by 52% in patients undergoing orthopedic surgery^13^. Meta-analyses of several randomized clinical trials have shown that the administration of TXA reduces blood loss and the blood transfusion rate without risk of thromboembolic events in patients undergoing THA and TKA^13–15^. The beneficial effects of TXA have also been demonstrated in spinal surgery of both adult and pediatric patients^16,17^.

Among orthopedic trauma literature, routine use of TXA during fracture fixation has been suggested; unfortunately, the majority of these studies focuses on hip fractures and do not address pelvic or acetabular injuries^11,18^. However, some authors have demonstrated the efficacy and safety of TXA in reducing perioperative blood loss^12,19^. One randomized clinical trial in 88 acetabular fracture patients analyzed the use of TXA and failed to find a difference in transfusion rates and average blood loss^20^. This current study aimed to compare the incidence of perioperative blood loss and transfusion between two groups of patients in which one group (group II), considered the experimental group, received perioperative administration of TXA while the other group (group I) did not receive any antifibrinolytic agent.

## Materials and methods

In this retrospective study, conducted from January 2010 to December 2021, data of 312 patients admitted and diagnosed with pelvic and acetabular fractures at level 1 trauma center of our institution, who were managed operatively by open reduction and internal fixation were included. Before the year 2016, TXA administration was not used in our trauma team as a routine perioperative prophylactic blood loss management plan. However, this protocol became effective in all operated patients from the year 2016; therefore, for the purpose of this study, patients were divided into two groups: group I (from January 2010 to December 2015), a total of 157 patients (91 males and 66 females) who were treated without perioperative TXA and group II (from January 2016 to December 2021), a total of 149 patients (81 males and 68 females), who received perioperative TXA. The study excluded six patients with incomplete data; the other patients excluded from this study were: patients with known contraindication to TXA such as allergy to TXA, active thromboembolic disorders, intracranial hemorrhage and history of DVT, all patients whose fractures were treated conservatively with external fixation, those managed using minimally invasive sacro-iliac screw fixation technique, those whose data was lost during the screening process (including patients who did not complete their outpatient clinic follow-up in our institution) and patients with less than three months follow-up. Any postoperative complications that occurred within the first three months follow-up were all recorded and analyzed.

Routinely, patients were considered suitable for open operative procedure if the hemoglobin level was not less than 10 g/dl. Patients were considered as potential candidates for postoperative blood transfusion when the hemoglobin level was lower than 7 g/dl, meanwhile the patients who manifested obvious symptoms of anemia due to an acute blood loss were also transfused. All patients received intravenous low molecular weight heparin in prevention of DVT (0.2–0.4 mg/kg of body weight depending on the individuals’ body weight); anticoagulation prophylaxis was continued for three to four weeks following the patients’ hospital discharge.

All patients in group II received preoperative 15 mg/kg of TXA (maximum 2 g) in 200 ml normal saline prior to the incision, usually within 30 min at the same time as the prophylactic antibiotic infusion. If intraoperative excessive bleeding was noticed, a topical use of TXA was frequently used. For operations that lasted for more than three hours, a repeated intravenous dose was required. Six hours following the surgery, an additional single dose was given. All operations were carried out by the same medical team and coauthors of the current study. The acetabular fractures when involving anterior column or both columns, were usually managed via Ilioinguinal (IL) or combined Stoppa plus Iliac fossa approach (S + IF); while fractures involving posterior wall (with significant displacement) required additional Kocher-Langenbeck (K-L). All patients were operated under general anesthesia, and post-operative drains were placed to monitor the amount of blood loss from the wound; drains were generally removed after 48 h or when the 24 h’s volume of fluid drainage did not exceed 50 ml.

The variable of interest in our study was the preoperative and postoperative first and third day hemoglobin and hematocrit levels, the total amount of blood loss, the transfusion rate and the amount of blood drained from the wound. The evaluation of the total amount of blood loss was done using the Nadler’s^21^ formula for blood volume and the calculation was done based on the hemoglobin balance using the equation previously used by Good et al.^22^.

Hbloss = BV × (Hbi ± Hbe)30.001 + Hbt (Hbloss = hemoglobin loss, BV = Blood volume, Hbi = preoperative Hemoglobin, Hbe = postoperative day 3 hemoglobin Hbt = amount of allogenic hemoglobin transfused); volume of Blood loss was calculated as follows: Blood loss = 1000 × Hbloss/Hbi; the described calculation method considers both the visible and the hidden blood loss in which the hidden blood was obtained by blood loss minus drainage blood.

The patients’ data was recovered from the hospital information system and recorded in a database created by the Excel 2010 program by Microsoft (Microsoft Corporation, Redmond, Washington, USA) for further processing. This data included: demographic variables such as age, sex, mechanism and site of injury; clinical data such as fracture type, concomitant injuries, management protocols and hospital length of stay (LOS) and the laboratory blood analyses such as hemoglobin level, hematocrit.

Descriptive statistics were performed to summarize the characteristics of the study group and subgroups, continuous variables were expressed as mean ± standard deviation (SD), categorical variables were displayed as counts (n) and percentages (%). The chi-square test and student *t* test were used to compare the differences between variables in the two groups, with a 95% confidence interval. All statistical analyses were done using SPSS version 20.0 (SPSS, IBM, USA).

### Ethics statement

This study was approved by the Institutional Review Board and the Ethics Committee of West China Hospital of Sichuan University (Date-number: 2019-1156). Since this study was conducted in accordance with the declaration of Helsinki and given the retrospective nature of the study, without the privacy of patients being breached, the informed consent was waived from the patients by the West China Hospital Ethic Committee.

## Results

A total of three hundred and six patients were enrolled in this study and divided into two groups of patients who did not receive perioperative TXA (157 patients in group I) and those who received perioperative TXA (a total of 149 patients in group II). The mean age was 46.21 ± 33 years-old in group I and 44.67 ± 16 years-old in group II. In both groups, men predominated, with 57.96% and 54.36% present respectively in group I and group II. Overall, there were no significant differences (all p > 0.5) in baseline parameters such as age, sex, mechanism of injury, BMI (body mass index), fracture pattern, operative approach or associated orthopedic injuries requiring operative management (Table 1).

**Table 1:** Demographics of patients and surgical characteristics.

	Group I	Group II	P
Number of patients	157	149	
Age (years)	46.21 ± 33	44.67 ± 16	0.31
Sex (percentage of male)	57.96%	54.36%	0.23
Mechanism of injury			
Fall from height, n (%)	21 (13.37%)	19 (12.75%)	0.37
Pedestrian hit by motor vehicle, n (%)	43 (27.38%)	46 (30.87%)	0.56
Motor vehicle collision n (%)	56 (35.66%)	53 (35.57%)	0.5
Motorcycle collision n (%)	30 (19.10%)	26 (17.44%)	0.31
Other n (%)	7 (4.45%)	5 (3.35)	0.47
BMI (mean ± SD)	17.48 ± 1.76	17.23 ± 1.84	0.35
Isolated pelvic ring fracture, n (%)	29 (18.47%)	23 (15.43%)	0.55
Isolated acetabular fracture, n (%)	84 (53.50%)	61 (40.93)	0.43
Combined pelvic and acetabular fracture, n (%)	44 (28.02%)	65 (43.62%)	0.11
Surgical approach			0.31
Anterior approach	116 (74.11%)	108 (72.48%)	
Posterior approach	25 (15.92%)	22 (14.76%)	
Combined anterior and posterior approach	16 (10.19%)	19 (12.75%)	
Concomitant orthopedic injuries managed operatively, n (%)	127 (80.89%)	122 (81.87%)	0.67

The average duration of operation was 259.3 ± 111.67 min in group I and 220 ± 101.31 min in group II (p = 0.09). The average preoperative hematocrit was higher in the preoperative period than in the postoperative first, second and third day in both groups (4.99% in group I and 2.0% in group II) and these values were slightly higher in the group of patients in which TXA was used; however, the difference was not significant. There was a significant difference in terms of estimated blood loss between the two groups, 1391 (± 167.49) ml in group I and 725 (± 403.31) ml in group II respectively (p = 0.02). The cell saver machine was used in 22.29% of patients in group I compared to 20.13% in group II. There was no significant difference in the total of intraoperative transfusion units as well as the total units of blood transfused. The analysis of hemoglobin level among the two studied populations revealed a reduced level of postoperative day 1 hemoglobin level (9.08 ± 1.72 g/dl in group I and 10.06 ± 2.75 g/dl in group II) compared to the values obtained in preoperative investigations (10.4 ± 2.37 g/dl in group I and 11.4 ± 2.08 g/dl in group II); comparable values were observed in day 2 and day 3, however, the differences remained non-significant. The results revealed a significant difference (p = 0.03) in the number of patients who were transfused following the operation with a percentage of 17.19% in group I versus 9.39% in group II. There was a significant difference in the volume of drained blood collected from the drains in both groups (p = 0.04). The study totalized 3 cases of symptomatic venous thromboembolisms, two in group I and one in group II. The major complications included respiratory failure, cardiac arrhythmia and renal failure; these complications did not show any significant difference in both groups (Table 2).

**Table 2:** Perioperative hematological data and complications.

	Group I	Group II	p
Duration of operation (minutes)	259.3 ± 111.67	220 ± 101.31	0.09
Preoperative hematocrit (percent)	34.22 (± 3.62)	33.44 (2.57)	0.12
Hematocrit postoperative day 1	27.31 ± 1.15	29.71 ± 5.64	0.14
Hematocrit postoperative day 2	27.01 ± 1.45	28.45 ± 6.77	0.23
Hematocrit postoperative day 3	29.23 ± 2.08	31.44 ± 6.31	0.37
Preoperative hemoglobin (g/dl)	10.4 ± 2.37	11.4 ± 2.08	0.11
Postoperative day 1 hemoglobin	9.08 ± 1.72	10.06 ± 2.75	0.87
Postoperative day 2 hemoglobin	9.14 ± 1.43	9.45 ± 1.12	0.25
Postoperative day 3 hemoglobin	9.01 ± 0.90	10.56 ± 1.66	0.41
Average estimated blood loss (ml)	1391 (± 167.49)	725 (± 403.31)	0.02
Cell saver cases n (percent)	35 (22.29%)	30 (20.13%)	0.52
Intraoperative transfusion (units)	1.23 ± 1.672	2.05 ± 1.08	0.44
Total units of blood transfused (intraoperatively and postoperatively)	3.71 ± 1.46	1.12 ± 2.16	0.32
Postoperatively transfused patients n (percent)	27 (17.19%)	14 (9.39%)	0.03
Drained blood volume (mL)	208 ± 117	157 ± 76	0.04
Venous thrombo embolism cases	2	1	0.49
Major complications cases			0.32
Respiratory failure	0	0	
Cardiac arythmia	1	2	
Renal failure	0	0	
Death	0	1	

The postoperative platelet count demonstrated a significant difference between the two groups at 24 h following the operation (p = 0.01); the perioperative coagulation parameters showed no significant difference (p > 0.05) in preoperative prothrombin time, postoperative day 1 prothrombin time, postoperative day 1 INR and the preoperative and postoperative day 1 APTT (Table 3).

**Table 3:** Perioperative coagulation parameters.

	Group I	Group II	p
Platelets count postoperative day 1 (Plts/microliter of circulating blood)	197.75 ± 129.07	234.68 ± 116.75	0.01
Preoperative (PT) (in seconds)	12.16 ± 1.52	11.21 ± 09	0.14
Postoperative prothrombin time (PT) day 1	12.05 ± 1.71	12.78 ± 1.67	0.16
INR postoperative day 1	1.08 ± 0.14	1.25 ± 1.11	0.10
Preoperative APTT (in seconds)	29.46 ± 4.94	29.01 ± 4.56	0.50
APTT postoperative day 1 (in seconds)	28.35 ± 4.82	32.01 ± 7.43	0.07

*Plt* platelets, *PT* prothrombin time, *INR* international normalized ratio, *APTT* activated partial thromboplastin time.

## Discussion

In the current study, our data demonstrated that perioperative chemoprophylaxis administration of TXA was effective in decreasing the requirement for allogenic blood product transfusion, reducing total blood loss and limiting HCT drop in patients undergoing surgical management of pelvic and acetabular fractures. Moreover, there were no trends in increasing the incidence of thromboembolic events in patients who received TXA.

Studies across surgical disciplines have demonstrated that, allogenic blood transfusions are an independent risk factor for several post-transfusion complications such as acute lung injury, circulatory overload, transfusion related infections (HIV, Hepatitis), increased risk of postoperative infection and mortality^23–25^; additionally, blood transfusions have been proven to have negative immune-modulating effects by impairing T cell-mediated immunity^26,27^. Increased risk of mortality due to blood transfusion has been reported even after three months following surgery for hip fractures^28^. Due to extensive bleeding that occurs in patients with pelvic and acetabular fractures, surgeons across the world commonly require the use of massive blood transfusions to reverse this potentially life-threatening condition.

The pelvic and acetabular region involves complex anatomical structures embedded in complex venous plexuses, which become vulnerable in initial injuries and major surgical interventions, thus leading to important blood loss and a need for blood transfusion. The transfusion rates in isolated pelvic fractures, acetabular fractures and a combination of the two are estimated at 24%, 35% and 57% respectively, with a mean transfusion of 4.8 units^29^. In the conquest of minimizing intraoperative bleeding, TXA has emerged to be a reliable alternative; its role in preventing massive bleeding in pelvic and acetabular fractures has been a matter of controversy amongst orthopedic trauma specialists. In this study, we attempted to prove whether the perioperative administration of TXA could decrease the total estimated blood loss and the risk of massive blood transfusion. According to our findings, there were no significant differences in preoperative and postoperative hematocrit. However, the average drop in hematocrit level was higher in patients who did not receive TXA compared to patients who received perioperative TXA administration (Table 2); this difference demonstrated evidence of perioperative prophylactic administration of TXA decreasing blood loss and it’s negative effects in patients receiving surgical treatment for pelvic and acetabulum fractures. In contrast there was a significant difference in the estimated volume of blood loss between patients who received perioperative TXA and those who did not receive it.

There was a trend towards a decreased blood transfusion rate among TXA patients (group II), with a significant difference in the number of patients who were transfused in both groups. However, we are aware that this finding could be affected by the initial anemic conditions and the choice of the operative approach which plays a role in blood loss as patients who usually undergo anterior approach are exposed to important blood loss^30^. In previous meta-analyses, some authors concluded that the use of TXA was associated with a lower transfusion rate and reduced estimated blood loss without increasing the risk of VTE^11,18^; while Zufferey et al.^31^ in a randomized control study evaluating the role of TXA reported a non-significant trend towards an increased risk of DVT in patients undergoing hip surgery. The use of TXA in joint replacement surgeries (TKA and THA) have demonstrated to possess strong therapeutic and safety potentials in limiting the chances of massive blood loss, thus decreasing the need for blood transfusion while decreasing the VTE risks^32–34^.

In contrast to joint arthroplasty patients, trauma patients have a relatively higher risk of fatal pulmonary embolism due to prolonged immobilization both preoperatively and postoperatively and therefore special attention needs to be given to this group of patients. The incidence of VTE has been reported to be 61% without prophylaxis and 2–33% with prophylaxis in pelvic and acetabular fracture patients^35–37^. In addition, TXA while inhibiting fibrinolysis, may create a prothrombic state; which is concerning for many practitioners resulting in a reluctance to use TXA in orthopedic trauma patients^18^. In a study conducted by Gumustas et al.^19^, evaluating the efficacy and safety of intravenous TXA in pelvic and acetabular fractures, the authors concluded that, in addition to their effectiveness in reducing total blood loss, hemoglobin drop and transfusion rate, TXA use did not increase the risk of venous and pulmonary thromboembolism. In a larger sample size study of 20,221 patients over 40 countries, it was demonstrated that there was a significant decrease in all bleeding causes and bleeding associated mortality when using TXA in trauma patients^38^. A similar conclusion was reached by several other authors^20,39,40^. In this study, we found a decreased estimated volume of blood loss and a decreased rate of transfusion in group II in whom TXA was peri-operatively administered. Additionally, we did not find any connection between the use of TXA and the incidence of DVT and PE. This finding was consistent with the aforementioned studies.

A prolonged operative time has also been suggested as an important risk factor for postoperative infection^41^; therefore, reducing the operation duration could decrease the risk of mortality and morbidity. In this study, the average operation time in group I was obviously superior compared to group II; even though the difference was not significant (p = 0.09) it is important to consider this factor as a merit in the use of TXA.

Some previous studies have listed several contraindication to TXA administration such as known active thromboembolic disorders, intracranial hemorrhage, history of DVT and allergy to TXA^42^. Additionally, some literatures define a three hour window period between injury time and TXA administration initiation timing, after which, the use of TXA is not beneficial to the patient. According to these authors, administration of TXA beyond three hours is conducive in an increased risk of pulmonary embolism (PE) and the development of venous thromboembolism (VTE)^43^. In contrast, a study conducted by Roberts et al.^44^ revealed no evidence supporting that administration of TXA after three hours of injury could increase the risk of any adverse events or head trauma-related mortality. In addition, other studies reported no high risk of VTE, PE^45^, and bleeding disorders in patients receiving administration of TXA^46^. The current study did not find any increased risk of thromboembolic events and its subsequent clinical manifestations. Allergy events after TXA administration have been discussed in previous literature with known clinical symptoms ranging from mild (skin rash, itching and/or urticaria) to severe (wheezing, hypotension and anaphylactic shock)^47^; some cases have therefore been reported in some previous studies^48,49^; In the current study, we did not notice the above mentioned adverse reactions, rather nausea and vomiting were usually reported in some patients in whom TXA was systematically discontinued and no case of TXA-related anaphylactic reaction was identified in any of our patients.

The incidence of postoperative complications in our studied population remained relatively low. To the best of our knowledge, the role of TXA has not revealed any major postoperative complications such as cardiac events (myocardial infarction/cardiac arrhythmia), renal failure and respiratory complications. In a meta-analysis involving 842 hip fractures patients, Haj-Younes et al.^50^ did not find significant differences in terms of myocardial infarction between the TXA and placebo group. In the CRASH-2 trial, it was reported that 1.05% of patients experienced arterial occlusive events such as myocardial infarction or stroke. However, the difference remained non-significant between the TXA and placebo group^38^.

In our clinical experience, patients administered with TXA commonly present with gastrointestinal symptoms such as nausea and vomiting. These side effects were previously reported in several studies^42,51,52^. This has been one of our greatest concerns when using TXA in our patients. In such patients, we have alternatively replaced TXA with aminocaproic acid.

One limitation of this study is the short duration of the follow up period which may conceal several possible long term postoperative complications and the retrospective nature of this study is susceptible to bias. In this study, we chose to compare TXA group patients to non-TXA group patients taken from the same hospital; one of our greatest concerns during our study was related to the changes that might probably occur in the management protocol of patients in our department from year 2010 to year 2021. Finally, we believe that the amount of estimated blood loss in pelvic and acetabular fracture surgeries can be associated with the operative approach, the numbers of incisions and the variability of different injuries (poly-trauma such as femoral fractures in which high blood loss is predicted) that characterized the study groups. Therefore we estimate that these factors may have influenced the result of the total blood loss in our study.

## Conclusion

In conclusion, our study unequivocally confirms that the use of TXA effectively reduces the risk of intraoperative bleeding during open management of pelvic and acetabular fractures. It should therefore be included in routine use when managing these patients.

## Data Availability

The datasets used and/or analysed during the current study are available from the corresponding author upon reasonable request.
